# 1,4,8,11-Tetra­azonia­cyclo­tetra­decane tetra­kis­(hydrogensulfate)

**DOI:** 10.1107/S1600536813018953

**Published:** 2013-07-20

**Authors:** Salem Said, Noureddine Mhadhbi, Fadhel Hajlaoui, Thierry Bataille, Houcine Naïli

**Affiliations:** a Laboratoire Physico-chimie de l’État Solide, Département de Chimie, Faculté des Sciences de Sfax, Université de Sfax, BP 1171, 3000 Sfax, Tunisia; b Laboratoire Sciences Chimiques de Rennes (CNRS, UMR 6226), Université de Rennes 1, Avenue du Général Leclerc, 35042 Rennes CEDEX, France

## Abstract

In the title salt, C_10_H_28_N_4_^4+^·4HSO_4_^−^, the cation lies about an inversion center. In the crystal, O—H⋯O and N—H⋯O hydrogen bonds connect the anions and cations, forming a three-dimensional network.

## Related literature

For the chemistry and applications of macrocyclic polyamine ligands, see: Wainwright (2001 ▶); Lukes *et al.* (2001 ▶); Zhang *et al.* (2003 ▶); Liu (2004 ▶). For related structures, see: Melson (1979 ▶); Subramanian & Zaworotko (1995 ▶); Ferchichi *et al.* (2010 ▶); Pojarová *et al.* (2010 ▶).
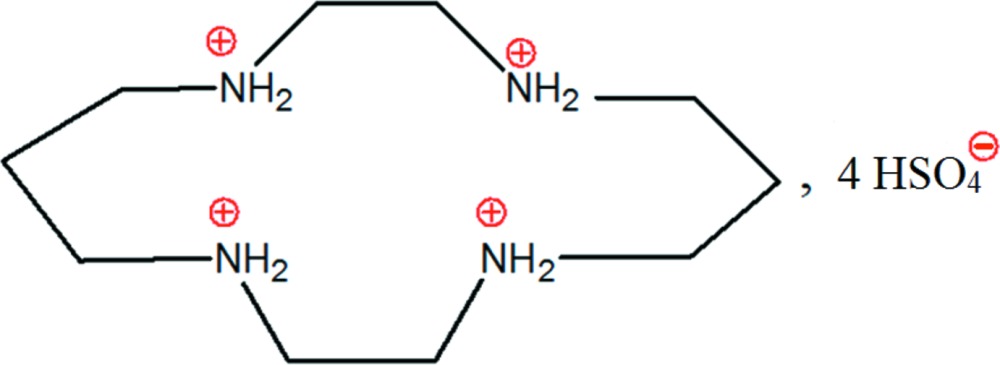


## Experimental

### 

#### Crystal data

C_10_H_28_N_4_^4+^·4HSO_4_^−^
*M* *_r_* = 592.68
Monoclinic, 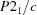

*a* = 7.8177 (2) Å
*b* = 16.6464 (3) Å
*c* = 8.7222 (2) Å
β = 97.165 (1)°
*V* = 1126.21 (4) Å^3^
*Z* = 2
Mo *K*α radiation
μ = 0.51 mm^−1^
*T* = 293 K
0.03 × 0.02 × 0.01 mm

#### Data collection

Nonius KappaCCD diffractometer
Absorption correction: analytical (face-indexed; de Meulenaer & Tompa, 1965 ▶) *T*_min_ = 0.988, *T*_max_ = 0.995
18757 measured reflections
4952 independent reflections
4074 reflections with *I* > 2σ(*I*)
*R*_int_ = 0.060

#### Refinement

*R*[*F* ^2^ > 2σ(*F* ^2^)] = 0.042
*wR*(*F* ^2^) = 0.123
*S* = 1.05
4952 reflections
163 parameters
H-atom parameters constrained
Δρ_max_ = 0.65 e Å^−3^
Δρ_min_ = −0.54 e Å^−3^

### 

Data collection: *COLLECT* (Nonius, 1998 ▶); cell refinement: *SCALEPACK* (Otwinowski & Minor, 1997 ▶); data reduction: *DENZO* (Otwinowski & Minor, 1997 ▶) and *SCALEPACK*; program(s) used to solve structure: *SHELXS97* (Sheldrick, 2008 ▶); program(s) used to refine structure: *SHELXL97* (Sheldrick, 2008 ▶); molecular graphics: *DIAMOND* (Brandenburg & Berndt, 1999 ▶); software used to prepare material for publication: *WinGX* (Farrugia, 2012 ▶).

## Supplementary Material

Crystal structure: contains datablock(s) global, I. DOI: 10.1107/S1600536813018953/lh5619sup1.cif

Structure factors: contains datablock(s) I. DOI: 10.1107/S1600536813018953/lh5619Isup2.hkl

Supplementary material file. DOI: 10.1107/S1600536813018953/lh5619Isup3.cml

Additional supplementary materials:  crystallographic information; 3D view; checkCIF report

## Figures and Tables

**Table 1:** Hydrogen-bond geometry (Å, °)

*D*—H⋯*A*	*D*—H	H⋯*A*	*D*⋯*A*	*D*—H⋯*A*
N1—H1*A*⋯O4^i^	0.90	2.60	3.2773 (18)	133
N1—H1*A*⋯O1^i^	0.90	1.97	2.8445 (14)	165
N1—H1*B*⋯O4	0.90	1.91	2.8051 (16)	171
N2—H2*A*⋯O3^ii^	0.90	2.02	2.8675 (15)	156
N2—H2*B*⋯O3^iii^	0.90	2.09	2.9117 (14)	151
N2—H2*A*⋯O7^iv^	0.90	2.45	2.9232 (15)	113
O2—H1⋯O8^i^	0.73	1.89	2.6125 (18)	172
O6—H2⋯O1^v^	0.91	1.85	2.7544 (17)	172

Symmetry codes: (i) 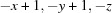; (ii) 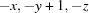; (iii) 
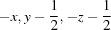
; (iv) 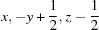; (v) 
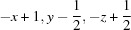
.

**Fig. 1.**
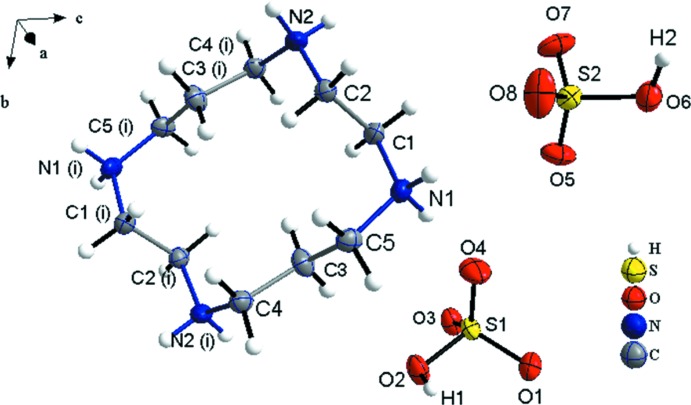
The molecular structure of the title compound, with the non-H atoms represented by 50% probability displacement ellipsoids; H atoms are shown as spheres of arbitrary radius [symmetry code: (i) -x, -y + 1, -z - 1].

**Fig. 2.**
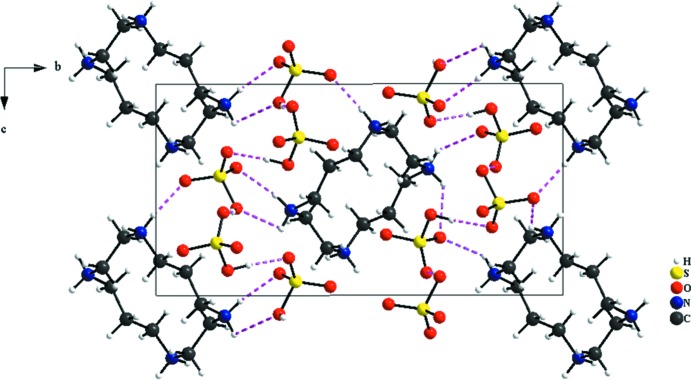
Projection of part of the crystal structure of the title compound along the a axis, with hydrogen bonds indicated as dashed lines.

## supplementary crystallographic information

### Comment

The chemistry of macrocyclic polyamine ligands with pendant arms has attracted much interest over the past two decades, because of their specific structures, chemical properties, their molecular recognition ability in the form of anions or cations, and their applications from radiopharmaceutical chemistry to waste-water treatment (Wainwright, 2001; Lukes *et al.*, 2001; Zhang *et al.*, 2003; Liu *et al.*, 2004). Herein we report preparation and crystal structure of the title compound.

The molecular structure of the title compound is shown in Fig. 1. The cation lies across a crystallographic inversion center and hence the asymmetric unit contains one half of the macrocyclic cation (cyclam) and two hydrogenosulfate anions. The tetra-protonated cyclam (C_10_H_28_N_4_)^4+^ cation exhibits C—C and C—N bond distances and angles in the range usually found for the cyclam molecule (Melson, 1979) and can be compared to related structures in the literature (Subramanian & Zaworotko, 1995; Ferchichi *et al.*, 2010; Pojarová *et al.*, 2010). In the crystal, O—H···O and N—H···O hydrogen bonds connect anions and cations to form a three-dimensional network (Fig. 2).

### Experimental

The title compound was prepared by mixing zinc(II) sulfate heptahydrate (1 mmol; 0.287 g), 1,4,8,11-tetraazoniacyclotetradecane (2 mmol; 0.400 g) and 20 ml water. The resulting solution was acidified with 1 ml concentrated sulfuric acid (1 mmol) under continuous stirring. The title compound was obtained accidentally as we intended to make a zinc complex. In 3 days, white crystals were formed. The synthesis is reproducible and crystals obtained in this way are stable for a long time under normal conditions of temperature and humidity. Single crystals of the title compound were grown by slow evaporation from the aqueous solution at room temperature.

### Refinement

H atoms bonded to C and N atoms were positioned geometrically and allowed to ride on their parent atom, with C—H = 0.97 Å, N—H = 0.90 Å and *U*_iso_ = 1.2*U*_eq_(C, N). H atoms bonded to O atoms were included in their 'as found' positions with refined isotropic displacement parameters.

### Crystal data

**Table:**

C_10_H_28_N_4_^4+^·4HSO_4_^−^	*F*(000) = 624.0
*M**_r_* = 592.68	*D*_x_ = 1.748 Mg m^−^^3^
Monoclinic, *P*2_1_/*c*	Mo *K*α radiation, λ = 0.71073 Å
Hall symbol: -P 2ybc	Cell parameters from 15635 reflections
*a* = 7.8177 (2) Å	θ = 2.9–35.0°
*b* = 16.6464 (3) Å	µ = 0.51 mm^−^^1^
*c* = 8.7222 (2) Å	*T* = 293 K
β = 97.165 (1)°	Prism, colourless
*V* = 1126.21 (4) Å^3^	0.03 × 0.02 × 0.01 mm
*Z* = 2	

### Data collection

**Table:**

Nonius KappaCCD diffractometer	4952 independent reflections
Radiation source: fine-focus sealed tube	4074 reflections with *I* > 2σ(*I*)
Graphite monochromator	*R*_int_ = 0.060
CCD rotation images, thick slices scans	θ_max_ = 35.0°, θ_min_ = 3.4°
Absorption correction: analytical (a face-indexed absorption correction was applied using the Tompa method; de Meulenaer & Tompa, 1965)	*h* = −12→10
*T*_min_ = 0.988, *T*_max_ = 0.995	*k* = −26→26
18757 measured reflections	*l* = −13→14

### Refinement

**Table:**

Refinement on *F*^2^	Secondary atom site location: difference Fourier map
Least-squares matrix: full	Hydrogen site location: inferred from neighbouring sites
*R*[*F*^2^ > 2σ(*F*^2^)] = 0.042	H-atom parameters constrained
*wR*(*F*^2^) = 0.123	*w* = 1/[σ^2^(*F*_o_^2^) + (0.0623*P*)^2^ + 0.4049*P*] where *P* = (*F*_o_^2^ + 2*F*_c_^2^)/3
*S* = 1.05	(Δ/σ)_max_ = 0.001
4952 reflections	Δρ_max_ = 0.65 e Å^−^^3^
163 parameters	Δρ_min_ = −0.54 e Å^−^^3^
0 restraints	Extinction correction: *SHELXL97* (Sheldrick, 2008), Fc^*^=kFc[1+0.001xFc^2^λ^3^/sin(2θ)]^-1/4^
Primary atom site location: structure-invariant direct methods	Extinction coefficient: 0.019 (6)

### Special details

**Table:**

Experimental. Data were corrected for Lorentz-polarization effects and an analytical absorption correction (de Meulenaer & Tompa, 1965) was applied. The structure was solved in the P 1 21/c 1 space group by the direct methods (S and O) and subsequent difference Fourier syntheses (all other atoms), with an exception for H atoms bonded to C and N atoms which are positioned geometrically.
Geometry. All e.s.d.'s (except the e.s.d. in the dihedral angle between two l.s. planes) are estimated using the full covariance matrix. The cell e.s.d.'s are taken into account individually in the estimation of e.s.d.'s in distances, angles and torsion angles; correlations between e.s.d.'s in cell parameters are only used when they are defined by crystal symmetry. An approximate (isotropic) treatment of cell e.s.d.'s is used for estimating e.s.d.'s involving l.s. planes.
Refinement. Refinement of *F*^2^ against ALL reflections. The weighted *R*-factor *wR* and goodness of fit *S* are based on *F*^2^, conventional *R*-factors *R* are based on *F*, with *F* set to zero for negative *F*^2^. The threshold expression of *F*^2^ > σ(*F*^2^) is used only for calculating *R*-factors(gt) *etc*. and is not relevant to the choice of reflections for refinement. *R*-factors based on *F*^2^ are statistically about twice as large as those based on *F*, and *R*- factors based on ALL data will be even larger.

### Fractional atomic coordinates and isotropic or equivalent isotropic displacement parameters (Å^2^)

**Table:**

	*x*	*y*	*z*	*U*_iso_*/*U*_eq_	
S1	0.34278 (4)	0.656923 (16)	0.06576 (3)	0.02143 (8)	
S2	0.23332 (4)	0.351066 (18)	0.25289 (4)	0.02525 (9)	
O1	0.49495 (13)	0.67391 (6)	0.17729 (12)	0.0309 (2)	
O2	0.37660 (15)	0.69761 (7)	−0.08875 (11)	0.0345 (2)	
O3	0.18911 (13)	0.69666 (7)	0.10348 (12)	0.0326 (2)	
O4	0.3244 (2)	0.57165 (7)	0.04142 (15)	0.0463 (3)	
O6	0.38228 (16)	0.32441 (7)	0.38175 (14)	0.0396 (3)	
O5	0.21214 (17)	0.43447 (6)	0.28810 (17)	0.0425 (3)	
O7	0.08566 (15)	0.30213 (7)	0.27346 (18)	0.0456 (3)	
O8	0.29980 (19)	0.33486 (11)	0.10785 (15)	0.0550 (4)	
N1	0.28066 (13)	0.46125 (6)	−0.20331 (12)	0.02426 (19)	
H1A	0.3615	0.4232	−0.1801	0.029*	
H1B	0.2844	0.4940	−0.1209	0.029*	
C1	0.10770 (16)	0.42072 (7)	−0.22403 (13)	0.0239 (2)	
H1C	0.0170	0.4609	−0.2348	0.029*	
H1D	0.0945	0.3882	−0.1340	0.029*	
N2	−0.08305 (14)	0.33181 (6)	−0.40184 (12)	0.02342 (19)	
H2A	−0.1098	0.3079	−0.3154	0.028*	
H2B	−0.0790	0.2932	−0.4735	0.028*	
C2	0.09283 (15)	0.36783 (7)	−0.36757 (13)	0.0224 (2)	
H2C	0.1175	0.3997	−0.4553	0.027*	
H2D	0.1777	0.3252	−0.3524	0.027*	
C4	−0.22592 (16)	0.38901 (7)	−0.45835 (14)	0.0239 (2)	
H4A	−0.2325	0.4310	−0.3821	0.029*	
H4B	−0.3349	0.3604	−0.4708	0.029*	
C5	0.33057 (15)	0.50958 (7)	−0.33674 (15)	0.0253 (2)	
H5A	0.3433	0.4739	−0.4226	0.030*	
H5B	0.4409	0.5353	−0.3062	0.030*	
C3	−0.19624 (18)	0.42678 (8)	−0.61163 (15)	0.0283 (2)	
H3A	−0.0825	0.4510	−0.6014	0.034*	
H3B	−0.2000	0.3852	−0.6899	0.034*	
H2	0.4133	0.2736	0.3570	0.103 (11)*	
H1	0.4645	0.6890	−0.1022	0.085 (10)*	

### Atomic displacement parameters (Å^2^)

**Table:**

	*U*^11^	*U*^22^	*U*^33^	*U*^12^	*U*^13^	*U*^23^
S1	0.02404 (14)	0.02050 (13)	0.01984 (13)	0.00254 (8)	0.00312 (9)	0.00086 (8)
S2	0.02107 (15)	0.02753 (15)	0.02768 (15)	0.00164 (9)	0.00516 (10)	−0.00477 (10)
O1	0.0275 (5)	0.0362 (5)	0.0274 (4)	0.0037 (4)	−0.0028 (3)	−0.0011 (4)
O2	0.0327 (5)	0.0474 (6)	0.0247 (4)	0.0048 (4)	0.0084 (4)	0.0110 (4)
O3	0.0251 (4)	0.0431 (5)	0.0307 (5)	0.0095 (4)	0.0082 (3)	0.0078 (4)
O4	0.0715 (9)	0.0225 (4)	0.0434 (6)	−0.0033 (5)	0.0013 (6)	−0.0054 (4)
O6	0.0375 (6)	0.0399 (6)	0.0382 (6)	0.0056 (4)	−0.0078 (4)	−0.0025 (5)
O5	0.0433 (6)	0.0233 (4)	0.0630 (8)	0.0016 (4)	0.0149 (5)	0.0004 (5)
O7	0.0285 (5)	0.0374 (6)	0.0720 (9)	−0.0091 (4)	0.0108 (5)	−0.0178 (6)
O8	0.0425 (7)	0.0928 (11)	0.0314 (6)	0.0243 (7)	0.0119 (5)	−0.0037 (6)
N1	0.0230 (4)	0.0228 (4)	0.0249 (4)	0.0006 (3)	−0.0051 (3)	−0.0002 (3)
C1	0.0255 (5)	0.0250 (5)	0.0204 (4)	−0.0032 (4)	0.0001 (4)	−0.0003 (4)
N2	0.0273 (5)	0.0176 (4)	0.0246 (4)	−0.0037 (3)	0.0001 (3)	0.0006 (3)
C2	0.0225 (5)	0.0211 (4)	0.0230 (5)	0.0002 (3)	0.0009 (4)	0.0000 (4)
C4	0.0219 (5)	0.0232 (5)	0.0263 (5)	−0.0024 (4)	0.0019 (4)	0.0014 (4)
C5	0.0187 (5)	0.0236 (5)	0.0328 (6)	−0.0003 (3)	0.0007 (4)	0.0014 (4)
C3	0.0297 (6)	0.0284 (5)	0.0270 (5)	0.0079 (4)	0.0046 (4)	0.0049 (4)

### Geometric parameters (Å, º)

**Table:**

S1—O4	1.4399 (11)	C1—H1D	0.9700
S1—O3	1.4449 (10)	N2—C2	1.4957 (15)
S1—O1	1.4672 (10)	N2—C4	1.5033 (16)
S1—O2	1.5601 (10)	N2—H2A	0.9000
S2—O5	1.4360 (11)	N2—H2B	0.9000
S2—O7	1.4423 (12)	C2—H2C	0.9700
S2—O8	1.4516 (13)	C2—H2D	0.9700
S2—O6	1.5779 (11)	C4—C3	1.5211 (17)
O2—H1	0.7258	C4—H4A	0.9700
O6—H2	0.9134	C4—H4B	0.9700
N1—C1	1.5017 (16)	C5—C3^i^	1.5199 (17)
N1—C5	1.5058 (17)	C5—H5A	0.9700
N1—H1A	0.9000	C5—H5B	0.9700
N1—H1B	0.9000	C3—C5^i^	1.5199 (17)
C1—C2	1.5232 (16)	C3—H3A	0.9700
C1—H1C	0.9700	C3—H3B	0.9700
			
O4—S1—O3	114.47 (8)	C4—N2—H2A	108.3
O4—S1—O1	110.22 (7)	C2—N2—H2B	108.3
O3—S1—O1	112.91 (6)	C4—N2—H2B	108.3
O4—S1—O2	108.98 (7)	H2A—N2—H2B	107.4
O3—S1—O2	103.49 (6)	N2—C2—C1	111.72 (10)
O1—S1—O2	106.16 (6)	N2—C2—H2C	109.3
O5—S2—O7	113.84 (7)	C1—C2—H2C	109.3
O5—S2—O8	115.43 (9)	N2—C2—H2D	109.3
O7—S2—O8	112.49 (9)	C1—C2—H2D	109.3
O5—S2—O6	102.36 (8)	H2C—C2—H2D	107.9
O7—S2—O6	106.48 (8)	N2—C4—C3	111.19 (10)
O8—S2—O6	104.82 (7)	N2—C4—H4A	109.4
S1—O2—H1	108.7	C3—C4—H4A	109.4
S2—O6—H2	106.6	N2—C4—H4B	109.4
C1—N1—C5	117.60 (9)	C3—C4—H4B	109.4
C1—N1—H1A	107.9	H4A—C4—H4B	108.0
C5—N1—H1A	107.9	N1—C5—C3^i^	111.43 (10)
C1—N1—H1B	107.9	N1—C5—H5A	109.3
C5—N1—H1B	107.9	C3^i^—C5—H5A	109.3
H1A—N1—H1B	107.2	N1—C5—H5B	109.3
N1—C1—C2	109.50 (10)	C3^i^—C5—H5B	109.3
N1—C1—H1C	109.8	H5A—C5—H5B	108.0
C2—C1—H1C	109.8	C5^i^—C3—C4	111.90 (11)
N1—C1—H1D	109.8	C5^i^—C3—H3A	109.2
C2—C1—H1D	109.8	C4—C3—H3A	109.2
H1C—C1—H1D	108.2	C5^i^—C3—H3B	109.2
C2—N2—C4	116.01 (9)	C4—C3—H3B	109.2
C2—N2—H2A	108.3	H3A—C3—H3B	107.9

Symmetry code: (i) −*x*, −*y*+1, −*z*−1.

### Hydrogen-bond geometry (Å, º)

**Table:**

*D*—H···*A*	*D*—H	H···*A*	*D*···*A*	*D*—H···*A*
N1—H1*A*···O4^ii^	0.90	2.60	3.2773 (18)	133
N1—H1*A*···O1^ii^	0.90	1.97	2.8445 (14)	165
N1—H1*B*···O4	0.90	1.91	2.8051 (16)	171
N2—H2*A*···O3^iii^	0.90	2.02	2.8675 (15)	156
N2—H2*B*···O3^iv^	0.90	2.09	2.9117 (14)	151
N2—H2*A*···O7^v^	0.90	2.45	2.9232 (15)	113
O2—H1···O8^ii^	0.73	1.89	2.6125 (18)	172
O6—H2···O1^vi^	0.91	1.85	2.7544 (17)	172

Symmetry codes: (ii) −*x*+1, −*y*+1, −*z*; (iii) −*x*, −*y*+1, −*z*; (iv) −*x*, *y*−1/2, −*z*−1/2; (v) *x*, −*y*+1/2, *z*−1/2; (vi) −*x*+1, *y*−1/2, −*z*+1/2.
